# Proceedings of the Erie County Medical Society, Annual Meeting

**Published:** 1885-02

**Authors:** Edward Clark

**Affiliations:** Secretary


### Proceedings of the Erie County Medical Society, Annual Meeting.

The annual meeting of the Erie County Medical Society was held in the Young Men's Christian Association Building, Jan. 13, 1885. The President, Dr. J. C. Greene, called the meeting to order and the following members were present, viz.: Drs. Gay, Cronyn, Crego, Pryor, F. H. Potter, Root, Marcley, Andrews, Ring, Gregory, E. H. Long, W. D. Greene, Briggs, Frederick, Thornton, F. W. Abbott, Wetmore, F. F. Hoyer,

Nott, Storck, Lynde, Runner, Ballou, Hancock, Brecht, Coakley, Dagenais, B. L. Lothrop, J. H. Potter, Hopkins, B. P. Hoyer, Diehl, Davidson, Howe, Keene, Samo, Thompson, Berkes, Prince, Campbell, Daggett, W. W. Potter, Strong, Bissell, Bailey, Dambach, Geo. Abbott, Kamerling, Haberstro, Dorland, Grove, Hawkins, Rochester, Jackson, Fowler, Boardman, Earl, Meisburger, Macniel, Hebenstreit, S. S. Greene, Van Peyma, Hauenstein, Wilson, Warren, Walsh, Hubbell, Wyckoff, Cary, Barker and Clark, and, by invitation, Drs. Sheehan and D. W. C. Greene.

The minutes of the semi-annual meeting of June 10th were read and approved.

After the reading and adoption of the minutes, Dr. Gay moved a suspension of the rules so that the society could at once listen to the annual address of the President. Carried.

At the conclusion of the President's address, Dr. Geo. Abbott moved that a vote of thanks be tendered to the President for the able and instructive paper which he had presented for their consideration. Carried.

The Committee on Membership presented the following report, which was received, and recommendations contained therein adopted:

Your Committee on Membership would respectfully report that they have examined the credentials of the following named gentlemen, and find that the diploma of each was granted by a legally constituted medical college in good standing. We therefore recommend that the following named gentlemen be now admitted to full membership in this society, viz.: William Pask, James Porter, J. C. Parmenter, A. B. Wilson, William G. Ring, F. P. Vandenburg and F. W. Hinkel.

Concerning one of the above applicants your committee would offer the following:

*Resolved*, That the Erie County Medical Society learns, with regret, of the early and sudden demise of William Pask, M. D., who, at our last semi-annual meeting, made application for membership in this society.

*Resolved*, That the foregoing expression of sympathy and regret be entered upon the minutes of our society, and a copy thereof be transmitted to the family of the deceased.

Respectfully submitted,

E. T. DORLAND,
T. M. JOHNSON,
*Committee on Membership.*

The newly-elected members who were present were introduced to the society and requested to sign the by-laws. As many were absent, Dr. Storck moved that the Secretary be directed to notify members of their election, so that they could be present and sign the by-laws.

Carried.

Applications for membership were received from Drs. D. W. C. Greene, C. F. Howard, E. E. Johnston and Thomas J. G. Sheehan, which were referred to the Committee on Membership.

Under the head of " Reports of Committees and Officers," Dr. Gay, chairman of the committee appointed to secure rooms for the society's use, presented the following report, which was received and adopted:

Your committee, appointed at the last meeting to procure a suitable room for the accommodation of the society, recommend the hall in the Y. M. C. A. building. Annual rental will not exceed twenty, and may not be more than ten dollars.

C. C. F. GAY,
H. R. HOPKINS,
U. C. LYNDE,
*Committee.*

Dr. F. W. Abbott, the Treasurer of the society, presented his annual report, which was as follows:

FRANK W. ABBOTT, TREASURER, IN ACCOUNT WITH THE ERIE COUNTY MEDICAL SOCIETY.

| | | Dr. | Cr. |
|---|---|---|---|
| 1884. | | | |
| Jan. 8. | To Cash on hand | $ 7.82 | |
| " 8. | " Dues and initiation fees | 137.50 | |
| Apr. 7. | " " " " " | 5.00 | |
| " 8. | " " " " " | 50.00 | |
| June 10. | " " " " " | 40.50 | |
| Nov. 19. | " " " " " | 2.00 | |
| 1885. | | | |
| Jan. 1. | " Interest on deposits | 6.76 | |
| 1884. | | | |
| Jan. 8. | By Paid John Ferguson, Janitor | | $ 10.00 |
| " 9. | " " Peter Paul & Bro., printing, etc. | | 13.90 |
| Feb. 4. | " " H. R. Hopkins, State Society dues | | 25.00 |
| " 5. | " " Norman E. Mack, printing | | 25.25 |
| " 23. | " " Ed. Storck, Chairman Board of Censors | | 25.00 |
| Mar. 31. | " " Rent Cadet Parlors, Sept. 8th | | 3.00 |
| Apr. 16. | " " Illustrated History of Buffalo | | 17.00 |
| June 12. | " " Peter Paul & Bro., cards and printing | | 36.85 |
| July 21. | " " Flint & Dorr, insurance | | 3.00 |
| Nov. 6. | " " Rent of Y. M. C. A. Hall | | 10.00 |
| 1885. | | | |
| Jan. 5. | " " Rogers, Locke & Milburn, for legal opinion in regard to Charter of Niagara University | | 25.00 |
| Jan. 5. | " " Rogers, Locke & Milburn, services at the hearing before the Governor | | 50.00 |
| 1885. | | $249.58 | $244.00 |
| Jan. 12. | By Balance in treasury | | 5.58 |

Respectfully submitted,

F. W. ABBOTT, *Treasurer.*

On motion of Dr. F. H. Potter, the report of the Treasurer was received and referred to an Auditing Committee.

The Chair named as such committee, Drs. F. H. Potter, A. H. Briggs and U. C. Lynde.

The Librarian, Dr. J. B. Samo, submitted the following report, which, on motion of Dr. Ring, was received, adopted and placed on file:

Mr. President—The report of the Librarian showeth that the general condition of the library is good; that the additions thereto, since the last report, have been some volumes of the Syd. Society's publications, and two volumes of the "History of Buffalo and Erie County;" that the number of volumes drawn out have been few, the most reasonable inference from which would seem to be that members of the Erie County Medical Society have ample libraries of their own, or else that they are not aware of the many valuable works the society's library contains. The Librarian would urge upon the younger members, at least, the importance of improving their acquaintance with the library. The regulations in regard to the drawing of books are, as stated in the by-laws, that any member not indebted to the society and residing within ten miles of the library may have at any time two volumes drawn in his (or her) own name, and retain the same one month, and if residing more than ten miles from the library may retain the same three months.

The library is accessible every day, from two till ten P. M., at 396 Pearl street, corner of Chippewa.

All of which is respectfully submitted.

J. B. Samo, *Librarian.*

Dr. Storck, chairman of the Board of Censors, stated that the board had no written report to present. He reported orally that during the last six months the board had endeavored to prosecute physicians who were practicing in violation of the law in this county. In these efforts Dr. Storck stated that the board was indebted to its attorney and to Police Justice King, for their earnest and efficient co-operation in seeking out and punishing the offenders. He was sorry he could not say the same of the gentlemen connected with the District Attorney's office. He stated that there were still some illegal practitioners in the city and county, mostly females, who had not been prosecuted, because the board had not been able to procure proper and sufficient evidence against them. In regard to Mrs. Kimball's establishment, evidence sufficient to warrant proceeding against her had not been procured. Concerning Dr. Don's dispensary, he stated that it was in charge of two regular physicians, one a graduate of the Buffalo Medical College and a member of this society; consequently, the board had no right to molest them in their work.

On motion, the report was received and adopted.

The Auditing Committee presented the following report, which, on motion of Dr. F. H. Potter, was received, adopted, placed on file and the committee discharged:

Your committee appointed to examine the report of the accounts of the Treasurer of this society for the past year, would respectfully report that they find said accounts true and correct.

(Signed) F. H. Potter,
A. H. Briggs,
U. C. Lynde,
*Committee.*

No report was received from the Committee on Legislation.

There being no other reports of committees and officers, business was opened under the head of "Election of Officers," and Dr. Wm. Ring moved that this society now proceed to the election of President for the ensuing year. Carried.

The Chair appointed as tellers Drs. A. A. Hubbell and Mary Berkes.

Dr. Geo. Abbott moved that Dr. Berkes be empowered to cast the ballot of this society for Dr. J. B. Andrews for President. Carried.

One ballot was cast, which bore the name of Dr. J. B. Andrews. Dr. Andrews was therefore declared unanimously elected President for the ensuing year.

Dr. Storck moved to proceed to a formal ballot for Vice-President. Carried.

The tellers announced the result of the ballot as follows: Total number of votes cast 53, of which Dr. Dorland received 31, Dr. W. W. Potter 10, Dr. Hopkins 3, Dr. O'Brian 2, Dr.

Thompson 2, Dr. Van Peyma 1, Dr. Pryor 1, Dr. Strong 1, Dr. Clark 2. Dr. Dorland having received a majority of the votes cast was declared duly elected Vice-President for the ensuing year.

On motion of Dr. Dambach, Dr. Dorland's election was made unanimous.

The President directed the members of the society to prepare their ballots for Secretary, and declared that nominations were in order. Dr. Rochester presented the name of Dr. Wm. H. Thornton, which nomination was endorsed by Dr. Barker. Dr. Crego placed in nomination Dr. Edward Clark. A single ballot was taken, resulting as follows: Total number of votes cast 56, of which Dr. Thornton received 27 and Dr. Clark 29. Dr. Clark was therefore declared duly elected Secretary for the ensuing year. The election, on motion of Dr. Rochester, was made unanimous.

Dr. Howe moved that Dr. Hubbell be empowered to cast the ballot of this society for Dr. F. W. Abbott for Treasurer, which was carried. One ballot was cast, which bore the name of Dr. F. W. Abbott. Dr. Abbott was therefore declared unanimously elected Treasurer for the ensuing year.

Dr. Wm. Ring moved that Dr. Hubbell be empowered to cast the ballot of this society for Dr. J. B. Samo for Librarian, which was carried. One ballot was cast, which bore the name of Dr. J. B. Samo. Dr. Samo was declared unanimously re-elected Librarian of this society for the ensuing year.

Dr. Andrews moved that the present Committee on Membership, consisting of Drs. Dorland, Mynter and Johnson, be re-elected, substituting the name of Dr. Berkes for that of Dr. Dorland, who had been elected to the office of Vice-President. Carried.

Dr. Cronyn offered the following resolution and moved its adoption:

"That inasmuch as the Laws of 1880 did away with the powers and duties of the Board of Censors as heretofore existing, resolved, that said board be abolished."

Dr. Hopkins moved as an amendment that this society now proceed to elect a Board of Censors.

Dr. Warner moved as a further amendment that a nominating committee of three be appointed, which was lost.

Dr. Cary offered as an additional amendment that the tellers be empowered to cast the ballot of this society for the present Board of Censors.

Dr. Cronyn insisted that the society must proceed in the regular form, whereupon Dr. Cary withdrew his amendment. The vote recurring upon the original resolution, as amended by Dr. Hopkins, it was carried, and the President directed the members to prepare their ballots for Censors.

While the tellers were counting the ballots for Censors, Dr. Barker moved to open under the head of "Miscellaneous Business." Carried.

Under this head Dr. Geo. Abbott offered the following intended amendment to the by-laws, viz.: "That the order of business as given in Article IV., Section 1, of our by-laws, be so amended as to make No. 13, namely, the valedictory address of the retiring President, as No. 5 thereof, and that the numbering of the several orders be made to conform to such change of order."

Dr. Hopkins offered the following resolutions, which, on motion of Dr. Crego, were adopted as read:

WHEREAS, This, the Medical Society of the County of Erie, has, during the year last past, frequently placed upon record its appreciation of the importance of the matter of State license of practitioners of medicine, and

WHEREAS, This subject is now receiving the earnest attention of the medical profession of the State as represented by the Medical Society of the State of New York, therefore,

*Resolved*, That this society would respectfully represent to the Medical Society of the State of New York its confirmed sense and conviction of the importance of securing to the profession and the public the advantages of a State Licensing Board.

*Resolved*, That it is the further sense of this society that such State Licensing Board should be comprised of men having no connections with any medical institutions liable to conflict with the full and unbiased discharge of duty as State Examiners.

*Resolved*, That the foregoing be officially transmitted to the Medical Society of the State of New York.

Dr. Storck offered the following intended amendment to the by-laws:

"That Article II. of the by-laws, entitled Officers, be amended by striking out after the word Censors the words: 'The Censors to be severally designated and named in the ballot as follows: Examiner in anatomy, physiology and surgery; examiner in practice of medicine and obstetrics; examiner in chemistry and pharmacy; examiner in materia medica and botany; examiner in medical jurisprudence and pathology.'"

Dr. Abbott moved that the Treasurer be instructed to pay all bills and indebtedness incurred by this society. Carried.

Under this head Dr. Barker moved the adoption of the following amendments to the by-laws, which were offered by him at the semi-annual meeting in June:

To amend Section 1, of Article V., so that it shall read: "Every physician or surgeon residing in the county of Erie who may hereafter wish to become a member of this society, shall make his application to the Secretary, who shall present it at a regular meeting of the society. It shall be referred to the Committee on Membership, who shall report the result of their investigation, accompanied with a recommendation for or against the applicant at the next regular meeting of the society. The applicant shall be admitted by a majority-vote of the members present."

To amend Section 4, of Article V., by striking out the words, "pay the Treasurer one dollar," so that it shall read:

"SEC. 4. Every member, when admitted, shall sign the by-laws and be entitled to a certificate of membership."

To amend Section 5, of Article V., so that it shall read:

"SEC. 5. Applications for admission to the society shall be accompanied by the initiation fee of two dollars, and shall be presented at a regular meeting, but shall not be acted upon until the next regular meeting. The application shall be in the handwriting," etc.

To amend Section 6, of Article VI., so that it shall read:

"SEC. 6. The Committee on Membership shall carefully investigate, and, if possible, determine whether or not all applicants are of temperate habits, good moral character, and legally authorized to practice physic and surgery in this State, and report the result of their investigation at a regular meeting of the society."

The amendments were adopted as read.

Under this head Dr. J. D. Hill moved that a committee consisting of the President of the society and Drs. Barker and Hopkins, be appointed to revise the by-laws, and report at the next regular meeting of the society. Carried.

A communication from abroad relating to the welfare of the insane was read, which, on motion of Dr. Crego, was received and placed on file.

The tellers at this point announced that the following named gentlemen had received a majority of the votes cast for Censors, and they were declared duly re-elected a Board of Censors for the ensuing year, viz.: Drs. H. R. Hopkins, E. Storck, A. H. Briggs, P. W. Van Peyma and F. F. Hoyer.

Dr. Crego moved that the present Committee on Legislation, consisting of Drs. J. C. Greene, E. Storck, M. D. Mann, A. R. Davidson, A. H. Briggs, H. R. Hopkins and F. S. Crego, be continued as a Committee on Legislation for the ensuing year. Carried.

The President-elect, Dr. J. B. Andrews, was duly inducted into office, and thanked the society kindly for the great honor they conferred by electing him to preside over their deliberations.

He said he felt it to be no small honor to be called to preside over the third largest county society in the State, and that he would endeavor to discharge the duties devolving upon him as President in a fair and impartial manner.

There being no other business before the society, the meeting adjourned.

EDWARD CLARK, *Secretary.*

